# Analytic simulation of thermophoretic second grade fluid flow past a vertical surface with variable fluid characteristics and convective heating

**DOI:** 10.1038/s41598-022-09301-x

**Published:** 2022-03-31

**Authors:** Nehad Ali Shah, Se-Jin Yook, Oreyeni Tosin

**Affiliations:** 1 Department of Mechanical Engineering, Sejong University, Seoul, 05006 Korea; 2 School of Mechanical Engineering, Hanyang University, 222 Wangsimni-ro, Seongdong-gu, Seoul, 04763 Republic of Korea; 3 Department of Physical Sciences, Precious Cornerstone University, Ibadan, Nigeria

**Keywords:** Aerospace engineering, Mechanical engineering

## Abstract

The study considers the effect of thermophoresis particle deposition on the flow properties of second grade fluid with variable viscosity, variable thermal conductivity and variable concentration diffusivity subjected to a convective boundary condition. To further describe the transport phenomenon, the special case of assisting and opposing flows is explored. Using similarity transformations, the governing equations of the fluid model are transformed and parameterized into a system of nonlinear ordinary differential equations. The approximate analytic solution of a dimensionless system is obtained through the Optimal Homotopy Analysis Method (OHAM). It is observed that velocity and temperature distributions are decreasing functions of the second grade parameter for both assisting and opposing flows. When the thermophoretic parameter is increased, the concentration distributions at the first and fourth orders of chemical reaction decrease. For both opposing and assisting flows, velocity distributions are enhanced due to larger temperature-dependent viscous parameters.

## Introduction

The mode of heat transfer by which heat energy is transferred between a surface and a moving fluid at different temperature is known as convection. Convective heat transfer is referred to as dominant and special form of heat transfer in gases and liquids mainly because it involves the joint process of heat diffusion and heat transfer by bulk fluid flow. Depending on how the fluid flow is generated, convection can be classified as forced (assisted) or natural (free) convection. An external impetus, such as a fan, pump, or the wind, forces the fluid to flow across the surfaces in forced convection. On the other side convection is termed natural or free if the fluid motion is generated by buoyancy forces that are induced by density difference due to the temperature variation in the fluid. When the fluid is heated from lower layer during the boiling process, thermal expansion occurs and the hotter lower layer of the fluid becomes less dense. Because colder fluid is denser than hotter fluid, buoyancy causes the hotter, less dense part of the fluid to rise while the colder, denser part of the fluid descends and replaces the hotter fluid. When the bottom layer component of the fluid becomes heated and travels upward to be replaced by the cooler fluid, the convective cycle begins. Free convection heat transfer has a wide range of applications. It influences the operating temperatures of power generating and electronic devices, it is important in establishing temperature distributions within the building (Theodore et al*.*^1^). The Grashof number is a dimensionless parameter that regulates fluid flow in natural convection and is used in conjunction with heat and mass transfer. This dimensionless number is valuable because it represents the ratio of buoyancy force to fluid viscosity force exerted on a fluid (see^1^ for details). Furthermore, the Grashof number is crucial because buoyant forces are what causes natural convection as hot and cold fluids flow higher and downward and viscous force tries to halt it. Recently, Koriko et al.^2^ discussed free convection boundary layer flow of thixotropic fluid with special attention to active and passive controls of nanoparticles. They reported that Grashof number contributes to the augmentation of velocity of the fluid for both active and passive controls of nanoparticles. Likewise, Ramudu et al*.*^3^ studied free convective Casson nanofluid flow past a stretching sheet.

The study of non-Newtonian fluid has captured the interest of numerous researchers because of its importance in industry and technology. The models are primarily characterized as rate, differential and integral type fluids. Owing to the complexities working with non-Newtonian fluid, several authors have student different non-Newtonian fluid models under different perceptions. Hydromagnetic flow of Casson fluid with thermal radiation was presented by Ramudu et al. (2020)^3^. Kumar et al.^4^ discussed numerical approach of stream and energy transport in MHD dissipative hybrid ferrofluids. Sugunamma et al. examined MHD boundary layer flow of micropolar fluid across a coagulated sheet with non-Fourier heat flux model. Ramadevi et al.^5^ explored MHD mixed convective flow of micropolar fluid with modified Fourier’s heat flux model. Ferdows et al.^6^ considered boundary layer flow of hybrid nanofluid with exponential radiation and viscous dissipation effects. Recently, Mabood et al*.*^7^ reported approximate analytic solution of radiative reactive micropolar fluid flows towards moving flat plate. Rivlin and Ericksen^8^ introduced the Rivlin–Ericksen fluid of grade two or second grade which is capable of predicting typical stress effects and is an important class of non-Newtonian fluids. Shah et al.^9^ investigated the boundary layer flow of upper convected Maxwell fluid with nanoparticles along a vertical surface with magnetic field. Transient MHD double convection flow fractionalized second grade fluid was considered by Siddique et al.^10^. Ahamad et al.^11^ analyzed heat and mass transfer in MHD boundary layer flow of a second-grade fluid past an infinite vertical permeable surface. A few representative studies involving the flow of second grade fluid have been extensively reported in the works of Idowu et al.^12^, Akinbola and Okoya^13^ and Olanrewaju and Abbas^14^.

Thermophoresis of particles is defined as the movement of tiny particles in the direction of a diminishing temperature gradient. In other words, particles in a heated environment move to a colder environment during the process. Tyndal^15^ noticed a particle-free zone surrounding a heated surface in dusty air. Aitken^16^ demonstrated that the effect was caused by a higher bombardment of particles from molecules in the heated zone compared to the cool region. The gas molecules migrating from the hot side of the particles have a higher velocity than those migrating from the cold side in this event. The particles clash with the molecules more strongly as they move quicker. According to Hayat and Qasim^17^, this phenomena has several applications in aerosol technology, silicon thin film deposition, and radioactive particle deposition in nuclear reactor safety simulations. Therefore, the velocity of particles is called thermophoretic velocity and the force experienced by suspended particles owing to the temperature gradient is called thermophoretic force with the force's direction opposing the temperature gradient (Stanford^18^). Recently characteristics of thermophoresis and Brownian motion on radiative reactive micropolar fluid was explored by Mabood et al*.*^7^.

A chemical reaction occurs when one or more substances are transformed into other ones. Chemical reactions are an essential part of both technology and daily living. Chemical reaction-based processes include the combustion of fuels, the production of glass, the brewing of beer, and the production of wine. In many cases, the rate of chemical reactions is determined by the concentration of the species itself, and the order of chemical reactions with respect to a given substance (such as a reactant, catalyst, or product) is known as the index or exponent to which the concentration term in the rate equation is raised, McNaught and Wilkinson^19^. The order of chemical reactions is known to be influenced by a variety of factors, the most basic of which is the first-order reaction, in which the rate of reaction is proportional to the concentration of species, Kundu et al.^20^. According to Themelis^21^, the majority of chemical reactions represented in applications are first-order when the reaction rate is dependent on a single component and the exponent value is one. Rahman and Uddin^22^ presented boundary layer flow of nanofluid with variable chemical reaction in a radiative vertical plate. Effects of thermal slip and chemical reaction on free convective nanofluid from a horizontal plate was investigated by Alsenafi and Ferdows^23^. Panigrahi et al.^24^ discussed impact of chemical reaction on Mhd flow between vertical walls. Ferdows and Al-Mdallal^25^ investigated the impact of chemical reaction on boundary layer flow with heat and mass transfer over a linearly stretched sheet. They reported that when the order of the chemical reaction rises, the flow profiles increase, and as the Schmidt number and chemical reaction parameter increase, the flow profiles decline.

It is a reality that a little rise in temperature boosts transport phenomena by lowering viscosity across the momentum boundary layer, therefore altering the heat transfer rate at the wall. The fluid properties that are more responsive to a rise in temperature are viscosity and thermal conductivity. Nima et al.^26^ studied bioconvection flow of non-Newtonian fluid embedded in porous medium with variable properties. Magnetized nanofluid flow of ferromagnetic nanoparticles from parallel stretchable rotating disk with fluid properties is explored by Shamshiddin and Mohamed^27^. Ferdows et al.^6^ deliberated a free convective power-law with variable viscosity and thermal conductivity. Mhd non-Newtonian fluid flow past an exponentially stretching surface with variable thermal conductivity was investigated by Anantha et al.^28^. Variable thermal conductivity and heat source/sink effects on mhd boundary layer flow past a linearly stretching sheet was studied by Sharma and Singh^29^. Omowaye and Animasaun^30^ reported upper-convected Maxwell fluid flow over a melting surface in a hot environment subject to thermal stratification with variable thermo-physical properties.

The current work considers the analysis of the flow of a chemically reacting boundary layer fluid in a thermophoretic second-grade fluid with variable viscosity, variable thermal conductivity, and variable concentration diffusivity embedded in a porous medium. The approximate analytic solution is obtained through Optimal Homotopy Analysis Method. The effects of embedded flow controlling parameters on fluid velocity, temperature, concentration, and shear stress were shown and analyzed graphically.

## Mathematical formulation

The introduction of Second grade fluid is to illustrate certain nonlinear effects that cannot be explained by the classical theory of Navier–Stokes for Newtonian fluids (see Truesdel and Noll^31^, Dunn and Fosdick^32^. The constitutive law of incompressible homogeneous fluids of degree 2 is given by

1 $$\sigma = -pl+2\vartheta {A}_{1}+{\alpha }_{1}{A}_{2}+{\alpha }_{2}{A}_{1}^{2},$$

where $\sigma$ is the Cauchy tensor, $p$ is the pressure, $\vartheta$ is the viscosity ${\alpha }_{1},{\alpha }_{2}$ are the normal stress modulli, ${A}_{1}$ and ${A}_{2}$ are the first two Rivlin-Ericksen tensors defined as

2 $${A}_{1}\left(u\right)=\frac{1}{2}\left[\nabla u+ \nabla {u}^{T}\right], {A}_{2}=\frac{D{A}_{1}}{{D}_{t}}+{\left(\nabla u\right)}^{T}{A}_{1}+{A}_{1}\nabla \left(\nabla u\right),$$

3 $$\mathrm{and }\frac{D}{Dt} ={\partial }_{t}+u.\nabla$$

is the material derivative.

Dunn and Fosdick^32^ established that

4 $${\alpha }_{1}+{\alpha }_{2}=0, {\alpha }_{1}\ge 0.$$

Writing the equation $\frac{Du}{Dt}={u}_{t}+u.\nabla \mathrm{u }=\mathrm{div\sigma },$ one obtains the second grade fluid.

The problem under discussion with the Cartessian coordinates $(x, y,z)$, we assume the flow is steady, incompressible, $2$-dimensional. The velocity takes the form

5 $$\overrightarrow{q}=\left[u\left(x,y\right), v\left(x,y\right), 0\right].$$

**Figure 1:**
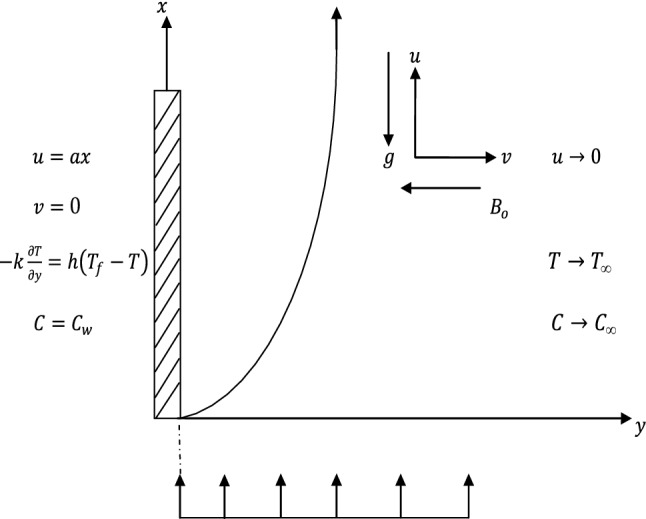
Physical model of the flow over a vertical surface.

The flow is taken along $x$-axis vertical and $y$-axis normal to it, as revealed by Fig. 1. It is assumed that the sheet is stretched with the linear velocity $u\left(x\right)=ax$, where $a>0$ is constant. The flow is subjected to convective heating process at its lower surface, which is characterized by a temperature ${T}_{f}$ and ${h}_{f}$ as a heat transfer coefficient and convective concentration near the surface is ${C}_{f}$. We assume ${C}_{w}$ is taken as the concentration at the surface, the *n*th order homogeneous chemical reaction with a rate constant. The temperature and concentration at the free stream are ${T}_{\infty }$ and ${C}_{\infty }$ respectively. The uniform magnetic field of magnitude ${B}_{o}$ is applied normal to the plate.

The continuity, momentum, energy, and concentration equations can be simplified using the usual boundary layer theory approximations for an incompressible fluid obeying the second grade fluid model with temperature dependent dynamic viscosity and thermal conductivity, as well as concentration dependent diffusivity following^13^

6 $$\frac{\partial u}{\partial x}+ \frac{\partial v}{\partial y}=0,$$

7 $$\begin{aligned} & u\frac{\partial u}{{\partial x}} + v\frac{\partial u}{{\partial y}} = \frac{1}{\rho }\frac{\partial }{\partial y}\left( {\mu \left( T \right)\frac{\partial u}{{\partial y}}} \right) - \frac{{\alpha_{1} }}{\rho }\left( {\frac{\partial }{\partial x}\left( {u\frac{{\partial^{2} u}}{{\partial y^{2} }}} \right) + \frac{\partial u}{{\partial y}}\frac{{\partial^{2} v}}{{\partial y^{2} }} + v\frac{{\partial^{3} u}}{{\partial y^{3} }} } \right) - \frac{\mu \left( T \right)}{\rho }\frac{1}{k}u \\ & \quad - \frac{{\sigma B_{o}^{2} u}}{\rho } + g\left[ {\beta_{T} \left( {T - T_{\infty } } \right) + \beta_{C} \left( {C - C_{\infty } } \right)} \right]. \\ \end{aligned}$$

Here the temperature dependent viscosity dynamical $(\mu )$ of the fluid can be expressed in the form Layek et al.^33^

8 $$\mu \left(T\right)={\mu }^{*}\left[1+\tau \left({T}_{f}-T\right)\right].$$

The basic boundary layer equation for the system of heat transfer in the presence of temperature dependent thermal conductivity exponential and space dependent heat source is expressed as

9 $$u\frac{\partial T}{\partial x}+ v\frac{\partial T}{\partial y}=\frac{1}{\rho {C}_{p}}\frac{\partial }{\partial y}\left(\kappa \left(T\right)\frac{\partial T}{\partial y}\right)+\frac{\kappa a}{\rho {C}_{p}\vartheta }\left[A\left({T}_{w}-{T}_{\infty }\right){e}^{-y{\left(\frac{a}{\vartheta }\right)}^{1/2}}+B\left(T-{T}_{\infty }\right)\right].$$

The mathematical model of temperature dependent thermal conductivity model of Charraudeau^34^, Salem and Fathy^35^ as

10 $$\kappa \left(T\right)={\kappa }^{*}\left[1+\delta \left(T-{T}_{\infty }\right)\right].$$

The boundary layer equation in conformity to the mass transfer in the occurrence of thermophoresis and chemical reaction is obtained in form^36^

11 $$u\frac{\partial C}{\partial x}+ v\frac{\partial C}{\partial y}=\frac{\partial }{\partial y}\left(D\left(C\right)\frac{\partial C}{\partial y}\right)-\frac{\partial }{\partial y}\left({V}_{T}C\right)-K{\left(C-{C}_{\infty }\right)}^{n}.$$

It will be valid to consider the model of variable concentration diffusivity given by the linear relation^37,38^

12 $$D\left(C\right)={D}^{*}\left[1+c\left(C-{C}_{\infty }\right)\right]={D}^{*}\left(1+b\varphi \right).$$

The thermophoretic velocity ${V}_{T}$ in Eq. (11) can be written in the form Talbot et al.^39^ as,

13 $${V}_{T}= -\frac{\kappa \vartheta \nabla T}{{T}_{ref}}= -\kappa \vartheta \frac{1}{{T}_{ref}}\frac{\partial T}{\partial y},$$

where $\kappa \vartheta$ represents the thermophoretic diffusivity, $\kappa$ is the thermophoretic coefficient which ranges in value from $0.2$ to $1.2$ as indicated by Batchelor and Shen^40^ and is defined from the theory of Talbot et al.^39^ which is given by

14 $$\kappa =\frac{2{C}_{s}\left(\frac{{\lambda }_{p}}{{\lambda }_{p}}+{C}_{t}Kn\right)\left[1+Kn\left({C}_{1}+{C}_{2}{exp}^{-\frac{{C}_{3}}{Kn}}\right)\right]}{\left(1+3{C}_{m}Kn\right)\left(1+\frac{2{\lambda }_{g}}{{\lambda }_{p}}+2{C}_{t}Kn\right)}.$$

Here, ${C}_{1}, {C}_{2}, {C}_{3}, {C}_{m}, {C}_{s} and {C}_{t}$ are constants, $\lambda$ and ${\lambda }_{p}$ are the thermal conductivities of the fluid and diffused particles respectively and $Kn$ is the Knudsen number. A thermophoretic parameter $\tau$ can be defined (see Refs.^41,36^) as,

15 $$\tau =\frac{\kappa \left({T}_{w}-{T}_{\infty }\right)}{{T}_{ref}}.$$

Equations (6), (7), (9) and (11) are subject to the following boundary conditions

16 $$u ={u}_{w}\left(x\right)=ax , v=0, -k\frac{\partial T}{\partial y}=h\left({T}_{f}-T\right), C={C}_{w} at y= 0,$$

17 $$u \to U\left(x\right)=0 , T \to {T}_{\infty } , C \to {C}_{\infty } as y\to \infty ,$$

where $u$ and $v$ are the velocity in $x$ and $y$ directions respectively, ${\mu }^{*}$ is the constant value of the coefficient, ${\kappa }^{*}$ is the coefficient thermal conductivity, ${D}^{*}$ is the coefficient concentration diffusivity, $\kappa$ is the thermal conductivity, $\rho$ is the density of the fluid second grade fluid. $\vartheta$ is the kinematic viscosity, $\mu =\vartheta \rho$ is the dynamic viscosity, $T$ is the fluid temperature in the boundary layer, $C$ is the concentration of the species diffusion, $D$ is the diffusion coefficient of the diffusing species, $\alpha =\frac{\kappa }{\rho {C}_{p}}$ is the thermal diffusivity.

The continuity Eq. (1) is satisfied by introducing a stream function $\psi$ such that

18 $$u=\frac{\partial \psi }{\partial y} , v= -\frac{\partial \psi }{\partial x}.$$

The Eqs. (7), (9), (11), (16) and (17) becomes

19 $$\begin{aligned} & \frac{\partial \psi }{{\partial y}}\frac{\partial }{\partial x}\frac{\partial \psi }{{\partial y}} - \frac{\partial \psi }{{\partial x}}\frac{\partial }{\partial y}\frac{\partial \psi }{{\partial y}} = \frac{1}{\rho }\frac{\partial }{\partial y}\left( {\mu \left( T \right)\frac{\partial }{\partial y}\left( {\frac{\partial \psi }{{\partial y}}} \right)} \right) - \frac{{\alpha_{1} }}{\rho }\left( {\begin{array}{*{20}c} {\frac{\partial }{\partial x}\left( {\frac{\partial \psi }{{\partial y}}\frac{{\partial^{2} }}{{\partial y^{2} }}\frac{\partial \psi }{{\partial y}}} \right) - } \\ {\frac{\partial }{\partial y}\frac{\partial \psi }{{\partial y}}\frac{{\partial^{2} }}{{\partial y^{2} }}\frac{\partial \psi }{{\partial x}} - \frac{\partial \psi }{{\partial x}}\frac{{\partial^{3} }}{{\partial y^{3} }}\frac{\partial \psi }{{\partial y}}} \\ \\ \end{array} } \right) \\ & \quad - \frac{{\sigma B_{o}^{2} }}{\rho }\frac{\partial \psi }{{\partial y}} - \frac{\mu \left( T \right)}{\rho }\frac{1}{k}\frac{\partial \psi }{{\partial y}} + g\beta_{T} \left( {T - T_{\infty } } \right) + g\beta_{C} \left( {C - C_{\infty } } \right), \\ \end{aligned}$$

20 $$\frac{\partial \psi }{\partial y}\frac{\partial T}{\partial x}-\frac{\partial \psi }{\partial x}\frac{\partial T}{\partial y}=\frac{1}{\rho {C}_{p}}\frac{\partial }{\partial y}\left(\kappa \left(T\right)\frac{\partial T}{\partial y}\right)+\frac{\kappa a}{\rho {C}_{p}\vartheta }\left[A\left({T}_{w}-{T}_{\infty }\right){e}^{-y{\left(\frac{a}{\vartheta }\right)}^\frac{1}{2}}+B\left(T-{T}_{\infty }\right)\right],$$

21 $$\frac{\partial \psi }{\partial y}\frac{\partial C}{\partial x}- \frac{\partial \psi }{\partial x}\frac{\partial C}{\partial y}=\frac{\partial }{\partial y}\left(D\left(C\right)\frac{\partial C}{\partial y}\right)-\frac{\partial }{\partial y}\left({V}_{T}C\right)-K{\left(C-{C}_{\infty }\right)}^{n}.$$

Subject to

22 $$\frac{\partial \psi }{\partial y}={u}_{w}\left(x\right)=ax, \frac{\partial \psi }{\partial y}=0, -k\frac{\partial T}{\partial y}=h\left({T}_{f}-T\right), C={C}_{w} at y=0,$$

23 $$\frac{\partial \psi }{\partial y}\to 0, T\to {T}_{\infty }, C\to {C}_{\infty } , as y\to \infty .$$

The momentum, energy and concentration equations can be transformed into corresponding ordinary differential equations by the following transformations

24 $$\eta =y\frac{{a}^\frac{1}{2}}{{\vartheta }^\frac{1}{2}}, \frac{\psi \left(x,y\right)}{x{a}^\frac{1}{2}{\vartheta }^\frac{1}{2}}=f\left(\eta \right), \theta \left(\eta \right)= \frac{T-{T}_{\infty }}{{T}_{f}-{T}_{\infty }}, \phi \left(\eta \right)= \frac{C-{C}_{\infty }}{{C}_{w}-{C}_{\infty }}.$$

Thus, $u$ and $v$ are given by $u=ax\frac{df}{d\eta }, v=-{a}^\frac{1}{2}{\vartheta }^\frac{1}{2}f\left(\eta \right),$ substituting (24) into Eqs. (19–23), we obtain

25 $$\begin{aligned} & [1 + \left( { 1 - \theta } \right)\xi \frac{{d^{3} f}}{{d\eta^{3} }} + f\left( \eta \right)\frac{{d^{2} f}}{{d\eta^{2} }} - \left( {\frac{df}{{d\eta }}} \right)^{2} - \xi \frac{{d^{2} f}}{{d\eta^{2} }}\frac{d\theta }{{d\eta }} - K\left( {2\frac{df}{{d\eta }} \frac{{d^{3} f}}{{d\eta^{3} }} - \left( {\frac{{d^{2} f}}{{d\eta^{2} }}} \right)^{2} - f\frac{{d^{4} f}}{{d\eta^{4} }}} \right) \\ & \quad - P_{s} \left[ {1 + \left( { 1 - \theta } \right)\xi } \right]\frac{df}{{d\eta }} - M\frac{df}{{d\eta }} + G_{r} \theta + G_{c} \phi = 0, \\ \end{aligned}$$

26 $$\left[1+\mathrm{\theta \varepsilon }\right]\frac{{\mathrm{d}}^{2}\uptheta }{{\mathrm{d\eta }}^{2}}-{\mathrm{P}}_{\mathrm{r}}\uptheta \frac{\mathrm{df}}{\mathrm{d\eta }}+{\mathrm{P}}_{\mathrm{r}}\mathrm{f}\left(\upeta \right)\frac{\mathrm{d\theta }}{\mathrm{d\eta }}+\upvarepsilon \frac{\mathrm{d\theta }}{\mathrm{d\eta }}\frac{\mathrm{d\theta }}{\mathrm{d\eta }}+\left[{\mathrm{Ae}}^{-\eta }+\mathrm{B\theta }\right]=0,$$

27 $$\left[1+\phi \omega \right]\frac{{d}^{2}\phi }{d{\eta }^{2}}+\omega {\left(\frac{d\phi }{d\eta }\right)}^{2}+{S}_{C}f\left(\eta \right)\frac{d\phi }{d\eta } -{S}_{c}\tau \left(\frac{d\theta }{d\eta }\frac{d\phi }{d\eta }+ \phi \left(\eta \right)\frac{{d}^{2}\theta }{{d\eta }^{2}}\right)-{S}_{c}\lambda {\phi }^{n}=0.$$

The corresponding boundary conditions take the form

28 $${f}^{^{\prime}}\left(\eta \right)=1, f\left(\eta \right)=0, \frac{d\theta }{d\eta }=-\gamma \left(1-\theta \left(\eta \right)\right), \phi \left(\eta \right)=1 at \eta =0,$$

29 $${f}^{^{\prime}}\left(\eta \right)\to 0 , \theta \left(\eta \right)\to 0, \phi \left(\eta \right)\to 0 as \eta \to \infty .$$

In the equations above $\eta$ is the independent dimensionless similarity variable, $A$ and $B$ are the heat source parameters, $K=\frac{{\alpha }_{1}a}{\rho \vartheta }$ is the second grade parameter, $M=\frac{\sigma {B}_{o}^{2}}{\rho a}$ is the magnetic field parameter, ${P}_{r}=\frac{\vartheta }{\alpha }$ is the Prandtl number, ${P}_{s}=\frac{\vartheta }{ka}$ is the porosity parameter, ${G}_{r}=\frac{g{\beta }_{T}({T}_{w}-{T}_{\infty })}{{a}^{2}x}$ is modified thermal Grashof number, ${G}_{c}=\frac{g{\beta }_{C}({C}_{w}-{C}_{\infty })}{{a}^{2}x}$ is modified solutal Grashof number, $\xi =b({T}_{f}-{T}_{\infty })$ is the temperature-dependent viscous parameter, $\varepsilon =\delta ({T}_{f}-{T}_{\infty })$ is the temperature dependent variable thermal conductivity parameter, $w=q({C}_{w}-{C}_{\infty })$ is the variable concentration diffusivity, $\lambda =\frac{{k}_{n}{\left({C}_{w}-{C}_{\infty }\right)}^{n-1}}{a}$ is the dimensionless chemical reaction parameter, ${S}_{c}=\frac{\vartheta }{D}$ is the Schimdt number.

## Optimal homotopy analysis solution

Invoking the rule of solution expressions above for ${f}_{o}\left(\eta \right), {\theta }_{o}\left(\eta \right)$ and ${\phi }_{o}(\eta )$ on (25–27) together with boundary conditions (28) and (29), the initial guesses ${f}_{o}(\eta )$, ${\theta }_{o}\left(\eta \right)$ and ${\phi }_{o}(\eta )$ which satisfies both the initial and the boundary conditions (28) and (29)

30 $${f}_{o}\left(\eta \right)=1-\mathrm{exp}\left(-\eta \right), {\theta }_{o}\left(\eta \right)=\frac{\gamma {e}^{-\eta }}{1+\gamma }, {\phi }_{o}\left(\eta \right)={e}^{-\eta }.$$

Linear operators ${L}_{f} , {L}_{p} and {L}_{\theta }$ are:

31 $${L}_{f} \left[f\left(\eta ,q\right)\right]=\frac{{\partial }^{3}f(\eta ;q)}{\partial {\eta }^{3}}-\frac{\partial f\left(\eta ;q\right)}{\partial \eta },$$

32 $${L}_{\theta } \left[\theta \left(\eta ,q\right)\right]=\frac{{\partial }^{2}\theta (\eta ;q)}{\partial {\eta }^{2}}-\theta \left(\eta ;q\right),$$

33 $${L}_{\phi } \left[\phi \left(\eta ,q\right)\right]=\frac{{\partial }^{2}\phi (\eta ;q)}{\partial {\eta }^{2}}-\phi \left(\eta ;q\right).$$

The operators ${L}_{f} , {L}_{p} and {L}_{\theta }$ have the following properties

$${L}_{f}[{C}_{1}+{C}_{2}\mathrm{exp}\left(-\eta \right)+{C}_{3}\mathrm{exp}\left(\upeta \right)]= 0, {L}_{p}[{C}_{4}\mathrm{exp}\left(-\eta \right)+{C}_{5}\mathrm{exp}\left(\upeta \right)]=0 ,$$

34 $${L}_{\theta }[{C}_{6}\mathrm{exp}\left(-\eta \right)+{C}_{7}\mathrm{exp}\left(\upeta \right)]=0.$$

In which ${C}_{1} , {C}_{2}$, ${C}_{3}$, ${C}_{4}$, ${C}_{5}$, ${C}_{6}$ and ${C}_{7}$ are arbitrary constants.

If $q\in [\mathrm{0,1}]$ denotes an embedding parameter, ${\hslash }_{f}, {\hslash }_{\theta }$ and ${\hslash }_{\phi }$ the non-zero auxiliary parameters then, the zeroth order of deformation problems are constructed as

35 $$\left(1-q\right){L}_{f}\left[f\left(\eta ;q\right)-{f}_{o}\left(\eta \right)\right]=q {\hslash }_{f}{H}_{f}\left(\eta \right)N\left[f\left(\eta ;q\right),p\left(\eta ;q\right), \theta \left(\eta ;q\right)\right],$$

36 $$\left(1-q\right){L}_{\theta }\left[\theta \left(\eta ;q\right)-{\theta }_{o}\left(\eta \right)\right]=q {\hslash }_{\theta }{H}_{\theta }\left(\eta \right)N\left[f\left(\eta ;q\right),p\left(\eta ;q\right), \theta \left(\eta ;q\right)\right],$$

37 $$\left(1-q\right){L}_{\phi }\left[\phi \left(\eta ;q\right)-{\phi }_{o}\left(\eta \right)\right]=q {\hslash }_{\phi }{H}_{\phi }\left(\eta \right)N\left[f\left(\eta ;q\right),p\left(\eta ;q\right), \phi \left(\eta ;q\right)\right].$$

Subject to boundary conditions

38 $$f\left(\eta =0;q\right)=0 , \frac{\partial f\left(\eta =0;q\right)}{\partial \eta }=1, \theta \left(\eta =0;q\right)=-\gamma \left[1-\theta \left(0;q\right)\right], \phi \left(\eta =0;q\right)=0,$$

39 $$\frac{\partial f\left(\eta \to \infty ;q\right)}{\partial \eta }\to 0 , \theta \left(\eta \to \infty ;q\right)\to 0, \phi \left(\eta \to \infty ;q\right)\to 0,$$

where the nonlinear operators are defined as

40 $$\begin{aligned} & N_{f} \left[ {f\left( {\eta ;q} \right), \theta \left( {\eta ;q} \right), \phi \left( {\eta ;q} \right)} \right] = [1 + \left( { 1 - \theta \left( {\eta ;q} \right)} \right)\xi \frac{{\partial^{3} f\left( {\eta ;q} \right)}}{{\partial \eta^{3} }} - f\left( {\eta ;q} \right)\frac{{\partial^{2} f\left( {\eta ;q} \right)}}{{\partial \eta^{2} }} \\ & \quad - \frac{{\partial^{2} f\left( {\eta ;q} \right)}}{{\partial \eta^{2} }} - \xi \frac{{\partial^{2} f\left( {\eta ;q} \right)}}{{\partial \eta^{2} }}\frac{{\partial \theta \left( {\eta ;q} \right)}}{\partial \eta } - K\left\{ {\begin{array}{*{20}c} {2\frac{{\partial f\left( {\eta ;q} \right)}}{\partial \eta }\frac{{\partial^{3} f\left( {\eta ;q} \right)}}{{\partial \eta^{3} }} - \left( {\frac{{\partial^{2} f\left( {\eta ;q} \right)}}{{\partial \eta^{2} }}} \right)^{2} } \\ { - f\left( {\eta ;q} \right)\frac{{\partial^{4} f\left( {\eta ;q} \right)}}{{\partial \eta^{4} }}} \\ \end{array} } \right\} \\ & \quad - M\frac{{\partial f\left( {\eta ;q} \right)}}{\partial \eta } + G_{r} \theta \left( {\eta ;q} \right) + G_{c} \phi \left( {\eta ;q} \right) = 0, \\ \end{aligned}$$

41 $$\begin{aligned} & N_{\theta } \left[ {f\left( {\eta ;q} \right), \theta \left( {\eta ;q} \right), \phi \left( {\eta ;q} \right)} \right] = [1 + \theta \left( {\eta ;q)} \right]\frac{{\partial^{2} f\left( {\eta ;q} \right)}}{{\partial \eta^{2} }} - P_{r} S_{t} \frac{{\partial f\left( {\eta ;q} \right)}}{\partial \eta } \\ & \quad - P_{r} \theta \left( {\eta ;q} \right)\frac{{\partial f\left( {\eta ;q} \right)}}{\partial \eta } + P_{r} f\left( {\eta ;q} \right)\frac{{\partial \theta \left( {\eta ;q} \right)}}{\partial \eta } + \varepsilon \frac{{\partial \theta \left( {\eta ;q} \right)}}{\partial \eta }\frac{{\partial \theta \left( {\eta ;q} \right)}}{\partial \eta } \\ & \quad + \left[ {{\text{Ae}}^{{ - {\text{y}}\left( {\frac{a}{\vartheta }} \right)^{\frac{1}{2}} }} + {\text{B}}\theta \left( {{\upeta };{\text{q}}} \right)} \right], \\ \end{aligned}$$

42 $$\begin{aligned} & N_{\phi } \left[ {f\left( {\eta ;q} \right), \theta \left( {\eta ;q} \right), \phi \left( {\eta ;q} \right)} \right] = [1 + \omega \phi \left( {\eta ;q)} \right]\frac{{\partial^{2} \phi \left( {\eta ;q} \right)}}{{\partial \eta^{2} }} + \omega \frac{{\partial \phi \left( {\eta ;q} \right)}}{\partial \eta }\frac{{\partial \phi \left( {\eta ;q} \right)}}{\partial \eta } \\ & \quad + S_{c} f\left( {\eta ;q} \right)\frac{{\partial \phi \left( {\eta ;q} \right)}}{\partial \eta } - S_{c} \tau \left( {\frac{{\partial \theta \left( {\eta ;q} \right)}}{\partial \eta }\frac{{\partial \phi \left( {\eta ;q} \right)}}{\partial \eta } + \phi \left( {\eta ;q} \right)\frac{{\partial^{2} \phi \left( {\eta ;q} \right)}}{{\partial \eta^{2} }}} \right) - S_{c} \lambda \phi \left( {\eta ;q} \right)^{n} , \\ \end{aligned}$$

where $q$ is an embedding parameter, ${\hslash }_{f}, {\hslash }_{\theta }$ and ${\hslash }_{\phi }$ are the non-auxiliary parameters and ${N}_{f}, {N}_{\theta }$ and ${N}_{\phi }$ are the non-linear operators. $q=0$ and $q=1$ we have;

$$f\left(\eta ;0\right)={f}_{o}\left(\eta \right), \theta \left(\eta ;0\right)={\theta }_{o}\left(\eta \right), \phi \left(\eta ;0\right)={\phi }_{o}\left(\eta \right)\mathrm{and}$$

43 $$f\left(\eta ;1\right)={f}_{o}\left(\eta \right), \theta \left(\eta ;1\right)={\theta }_{o}\left(\eta \right), \phi \left(\eta ;1\right)={\phi }_{o}\left(\eta \right)$$

and $f\left(\eta ;q\right), \theta (\eta ;q)$ and $\phi (\eta ;q)$ vary from ${f}_{o}\left(\eta \right), {\theta }_{o}\left(\eta \right)$ and ${\phi }_{o}(\eta )$ when $q$ varies from $0$ to $1$.

By Taylors series expansion one has;

44 $$f\left(\eta ;q\right)={f}_{o}\left(\eta \right)+ \sum_{m=1}^{\infty }{f}_{m}(\eta ){q}^{m},$$

45 $$\theta \left(\eta ;q\right)={\theta }_{o}\left(\eta \right)+ \sum_{m=1}^{\infty }{\theta }_{m}(\eta ){q}^{m},$$

46 $$\phi \left(\eta ;q\right)={\phi }_{o}\left(\eta \right)+ \sum_{m=1}^{\infty }{\phi }_{m}(\eta ){q}^{m}.$$

Clearly, the convergence of series Eqs. (35–37) is closely associated with ${\hslash }_{f}, {\hslash }_{\theta }$ and ${\hslash }_{\phi }.$ The auxiliary parameter ${\hslash }_{f}, {\hslash }_{\theta }$ and ${\hslash }_{\phi }$ are chosen such that the series Eqs. (35–37) converge at $q=1$. Hence,

47 $$f\left(\eta ;q\right)={f}_{o}\left(\eta \right)+ \sum_{m=1}^{\infty }{f}_{m}\left(\eta \right){q}^{m},$$

48 $$\theta \left(\eta ;q\right)={\theta }_{o}\left(\eta \right)+ \sum_{m=1}^{\infty }{\theta }_{m}\left(\eta \right){q}^{m},$$

49 $$\phi \left(\eta ;q\right)={\phi }_{o}\left(\eta \right)+ \sum_{m=1}^{\infty }{\phi }_{m}(\eta ){q}^{m}.$$

If we denote the special solution ${f}_{m}^{*}, {\theta }_{m}^{*}$ and ${\phi }_{m}^{*}$ then the general solutions ${f}_{m}, {\theta }_{m}$ and ${\phi }_{m}$ are.

50 $${f}_{m}\left(\eta \right)={f}_{m}^{*}\left(\eta \right)+{C}_{1}+{C}_{2} \mathrm{exp }\left(\eta \right)+{C}_{3} \mathrm{exp}\left(-\eta \right),$$

51 $${\theta }_{m}\left(\eta \right)={\theta }_{m}^{*}\left(\eta \right)+{C}_{4}+{C}_{5}\mathrm{exp}\left(\eta \right)+{C}_{6}\mathrm{exp}\left(-\eta \right),$$

52 $${\phi }_{m}\left(\eta \right)={\phi }_{m}^{*}\left(\eta \right)+{C}_{7}\mathrm{exp}\left(\eta \right)+{C}_{8}\mathrm{exp}\left(-\eta \right).$$

Here, ${f}_{m}^{*}\left(\eta \right), {\theta }_{m}^{*}\left(\eta \right)$ and ${\phi }_{m}^{*}\left(\eta \right)$ are the particular solutions of Eqs. (47–49)$.$ Following the rule of expression, the rule of coefficient ergodicity and the rule of solution existence as discussed in Liao^42^ we choose auxiliary functions as

53 $${H}_{f}={H}_{\theta }={H}_{\phi }=1.$$

## Convergence of the optimal homotopy solutions

It is obvious that the series (44–46) consist of the non-zero auxiliary parameters ${\hslash }_{f}, {\hslash }_{\theta }$ and ${\hslash }_{\phi }$ which can adjust and control the convergence. The interval on $\hslash$-curve becomes parallel to the $\hslash$-axis is recognized as the set of admissible values of ${\hslash }_{f}, {\hslash }_{\theta }$ and ${\hslash }_{\phi }$ for which the solutions series converges. In Figs. 16, 17 and 18, the range of the acceptable values of ${\hslash }_{f}, {\hslash }_{\theta }$ and ${\hslash }_{\phi }$ are $-0.75\le {\hslash }_{f}-0.35$, $-3.50\le {\hslash }_{\theta }-1.50$ and $-0.80\le {\hslash }_{f}-0.40$. Obviously, from the $\hslash$-curves for this problem, we obtained the approximate optimal values of ${\hslash }_{f}, {\hslash }_{\theta }$ and ${\hslash }_{\phi }$ at $10th$-order of approximations as $-1.2564, -0.3507$ and $-1.0344$

## Results and discussion

**Figure 2:**
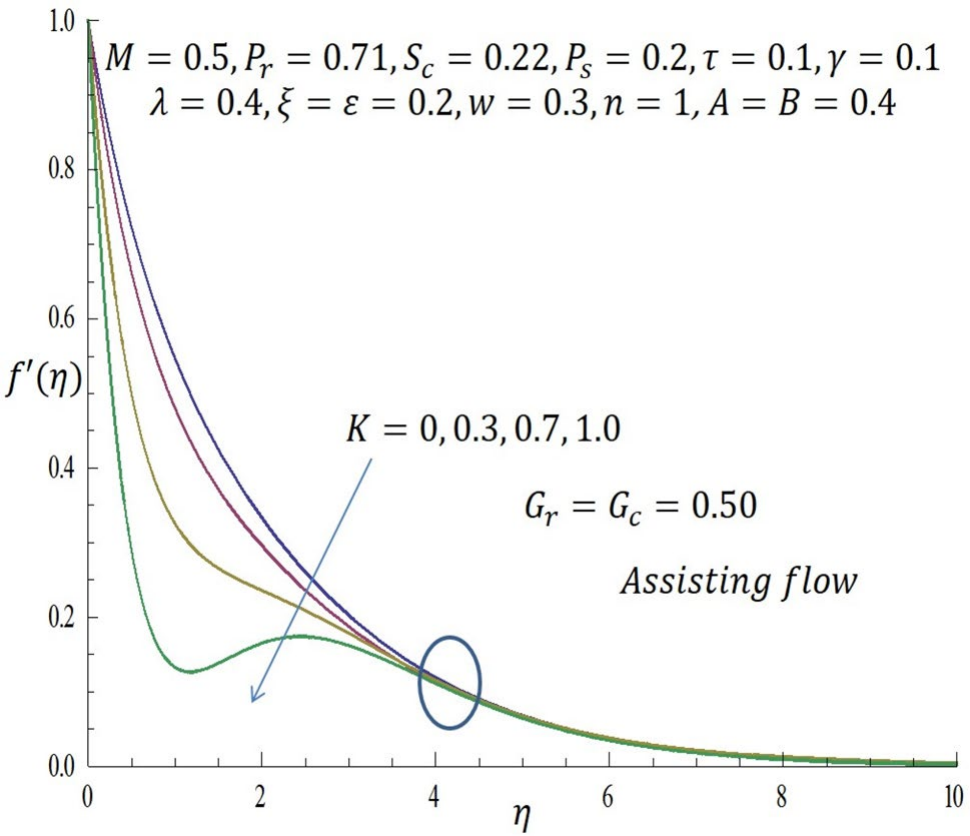
Effect of $K$ on velocity profile when *G*_*r*_ = *G*_*c*_ = 0.5.

**Figure 3:**
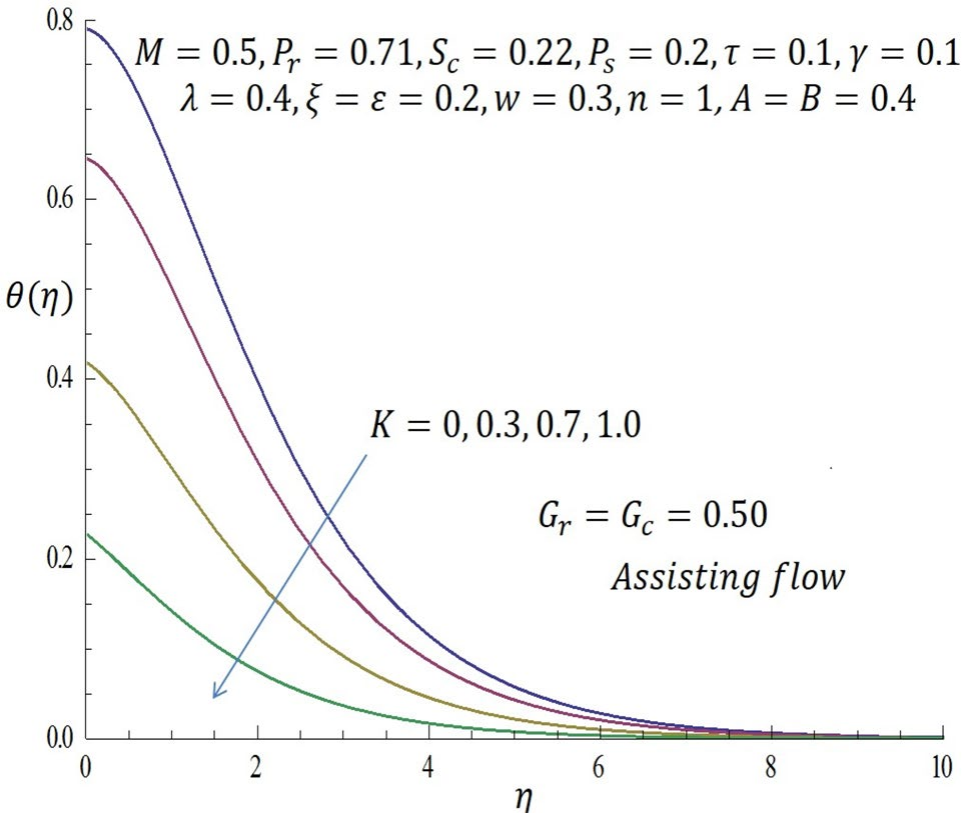
Effect of $K$ on temperature profile when *G*_*r*_ = *G*_*c*_ = 0.5.

**Figure 4:**
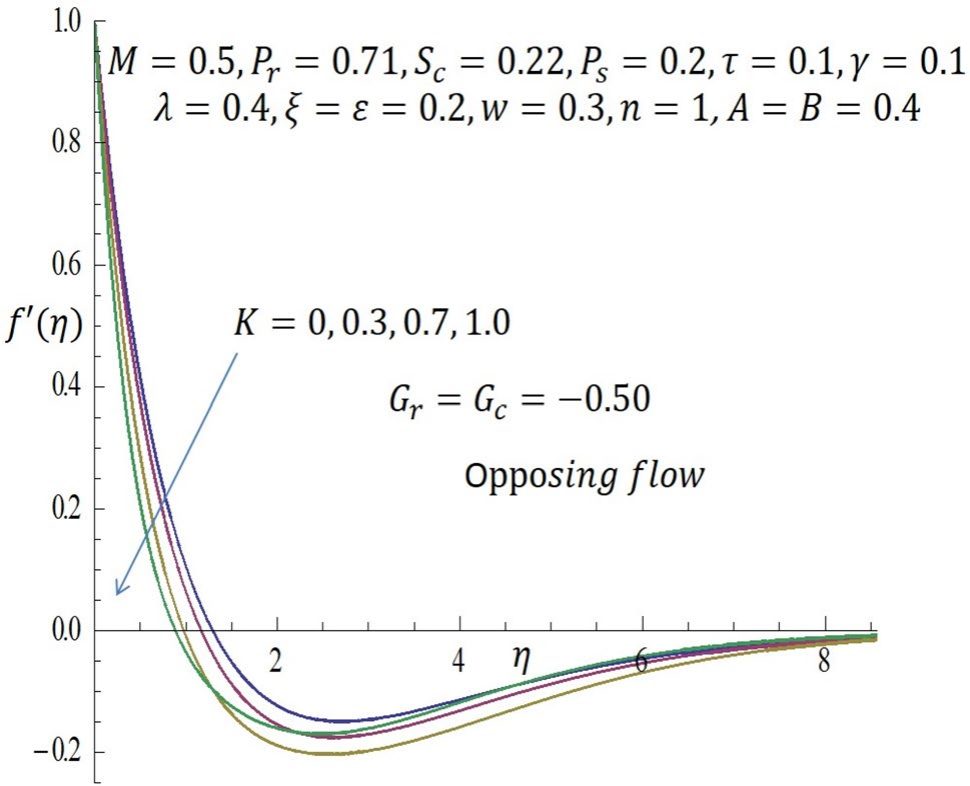
Effect of $K$ on velocity profile when ${G}_{r}={G}_{c}=-0.5$.

**Figure 5:**
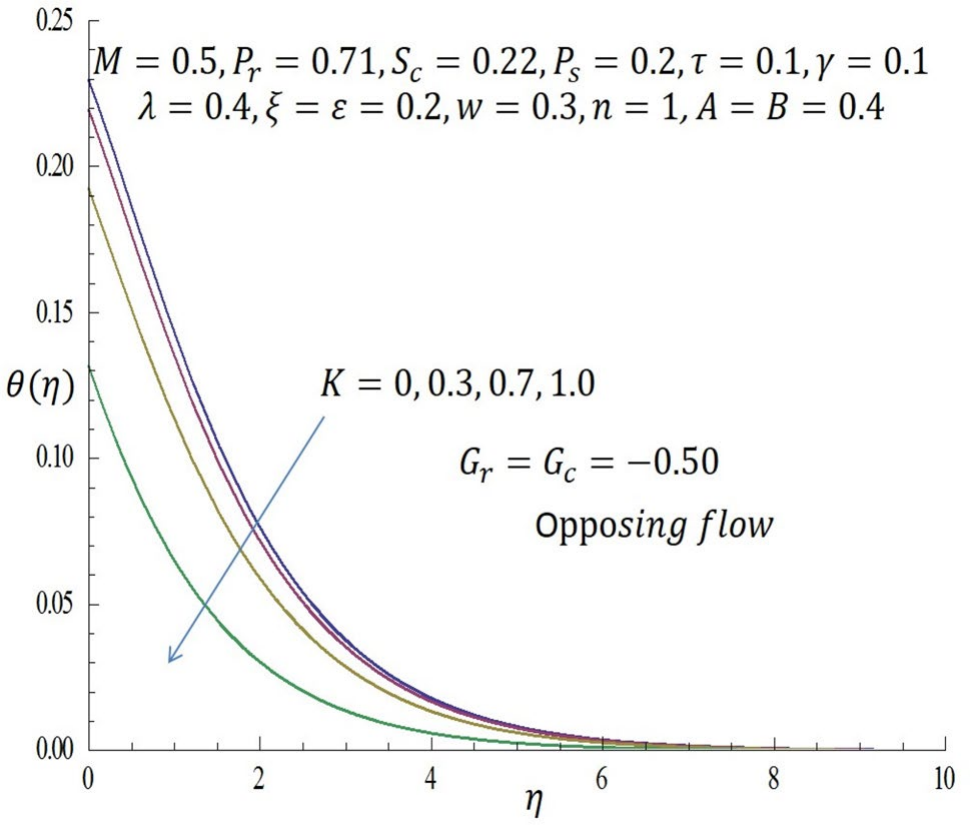
Effect of $K$ on temperatureprofile when ${G}_{r}={G}_{c}=-0.5.$

**Figure 6:**
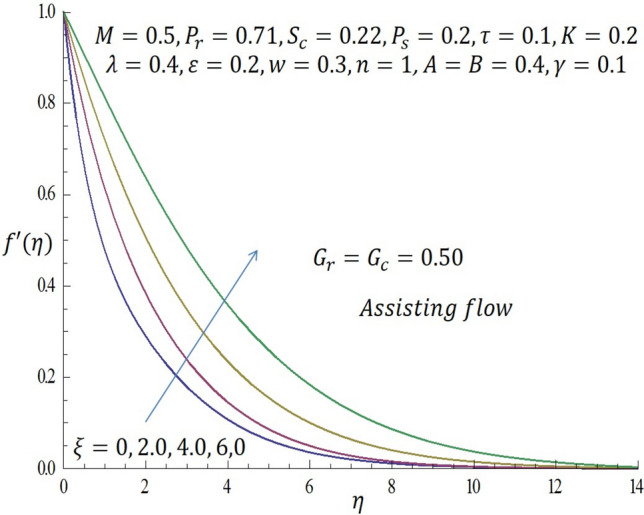
Effect of $\xi$ on velocity profile when ${G}_{r}={G}_{c}=0.5$.

**Figure 7:**
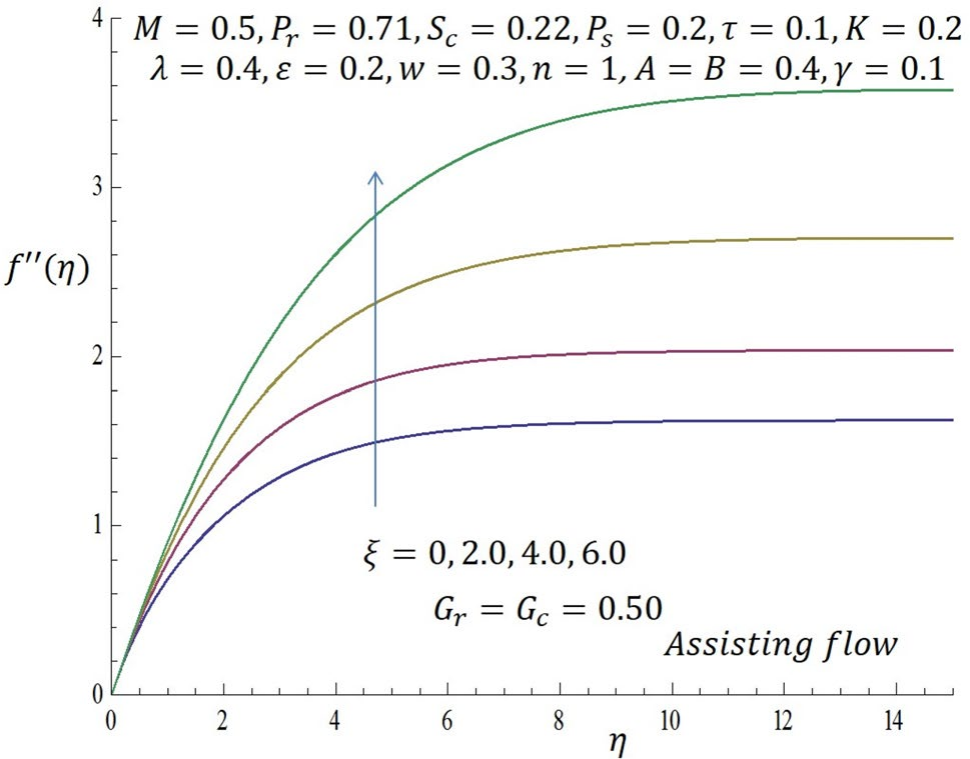
Effect of $\xi$ on shear stress profile when ${G}_{r}={G}_{c}=0.5.$

**Figure 8:**
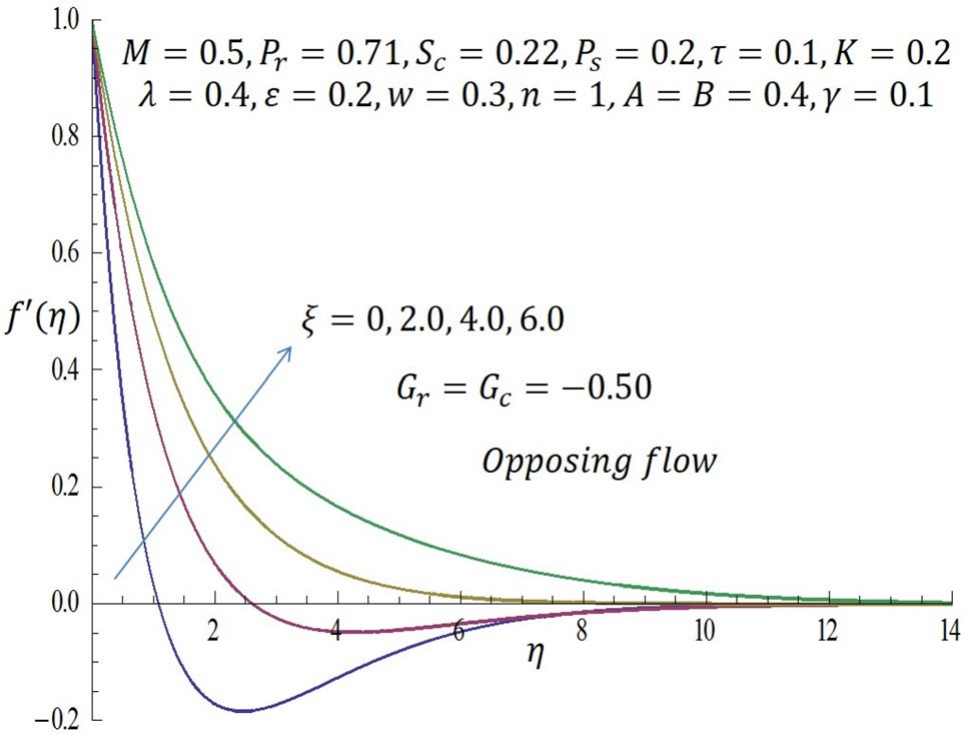
Effect of $\xi$ on velocity profile when ${G}_{r}={G}_{c}=-0.5$.

**Figure 9:**
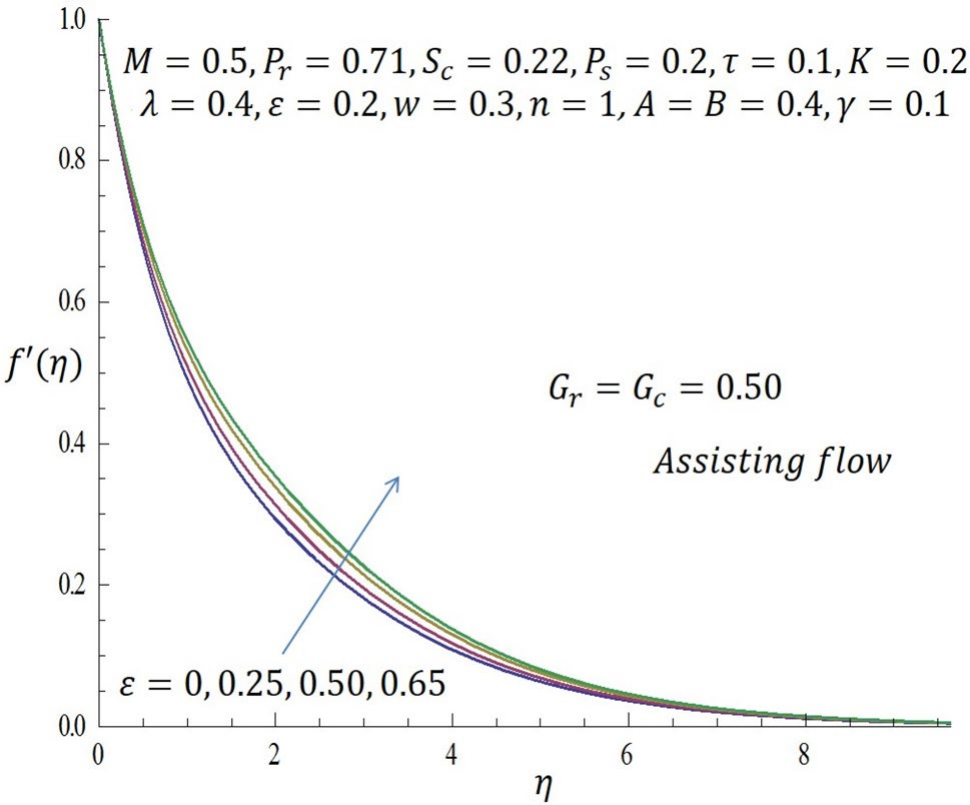
Effect of $\varepsilon$ on velocity profile when ${G}_{r}={G}_{c}=0.5$.

**Figure 10:**
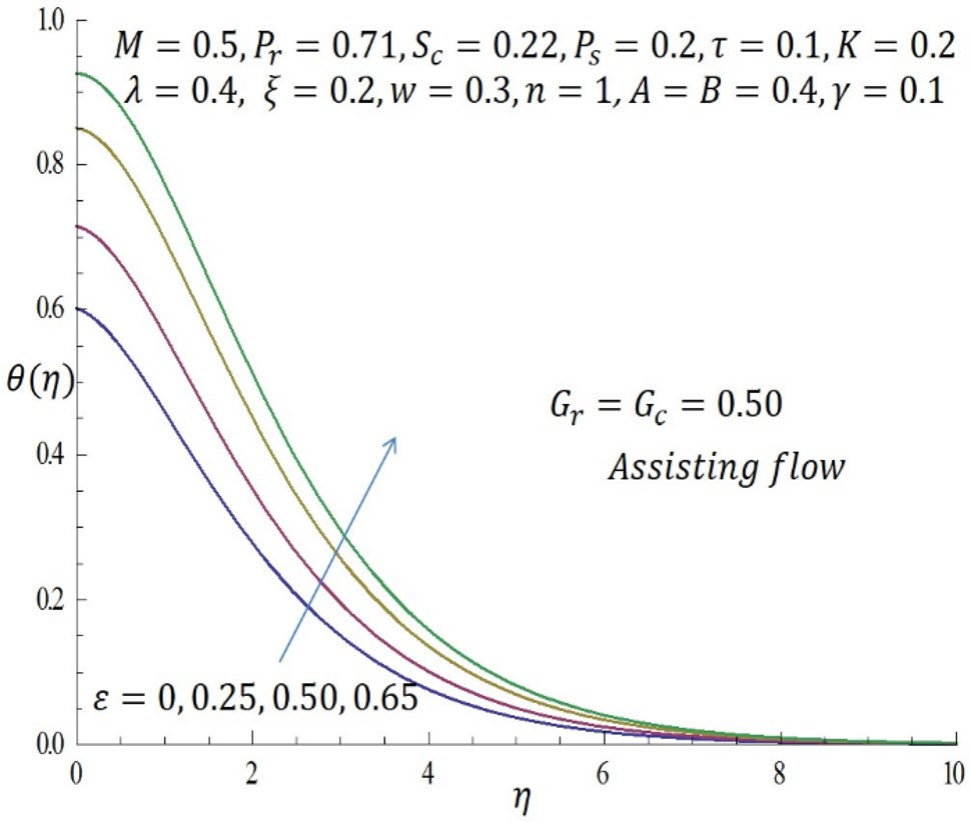
Effect of $\varepsilon$ on temperature profile when ${G}_{r}={G}_{c}=0.5.$

**Figure 11:**
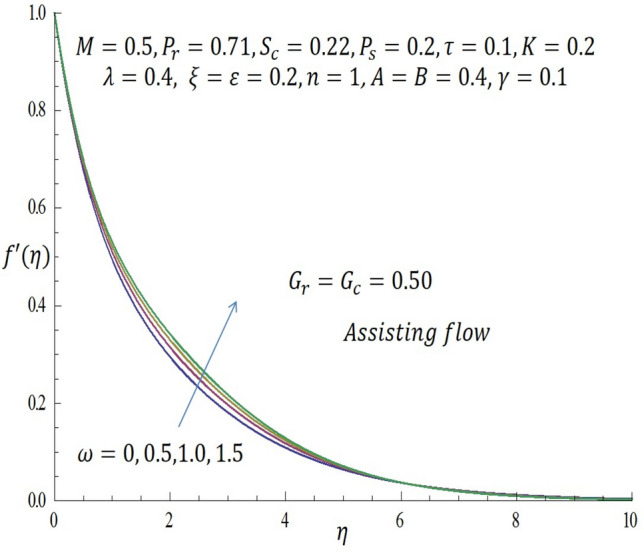
Effect of $\omega$ on velocity profile when ${G}_{r}={G}_{c}=0.5$.

**Figure 12:**
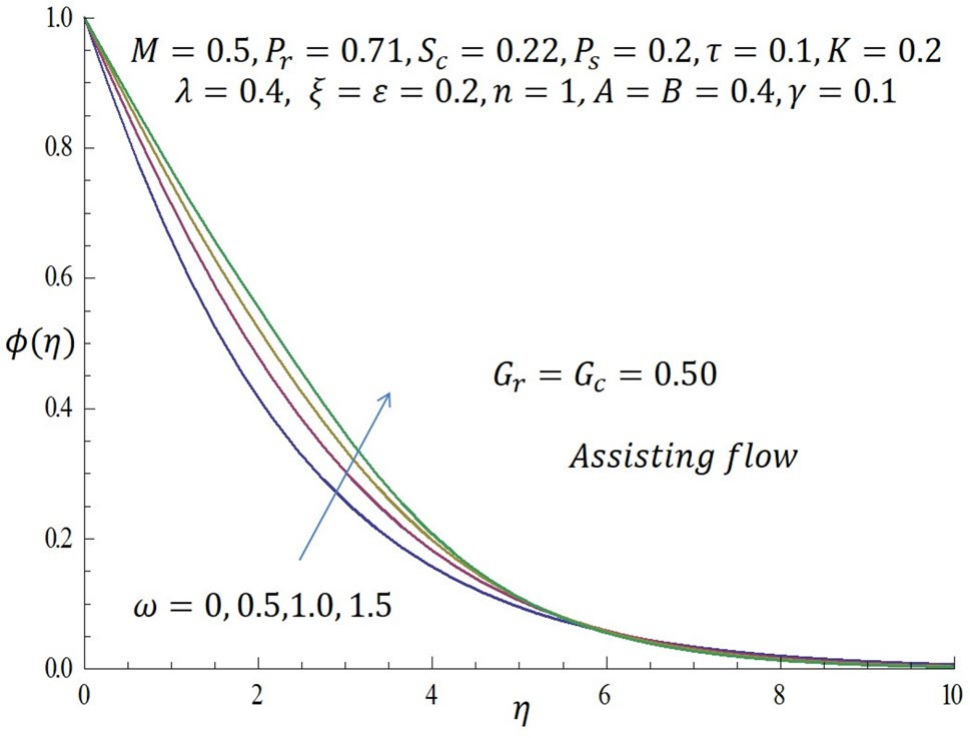
Effect of $\gamma$ on concentration profile when ${G}_{r}={G}_{c}=0.5.$

**Figure 13:**
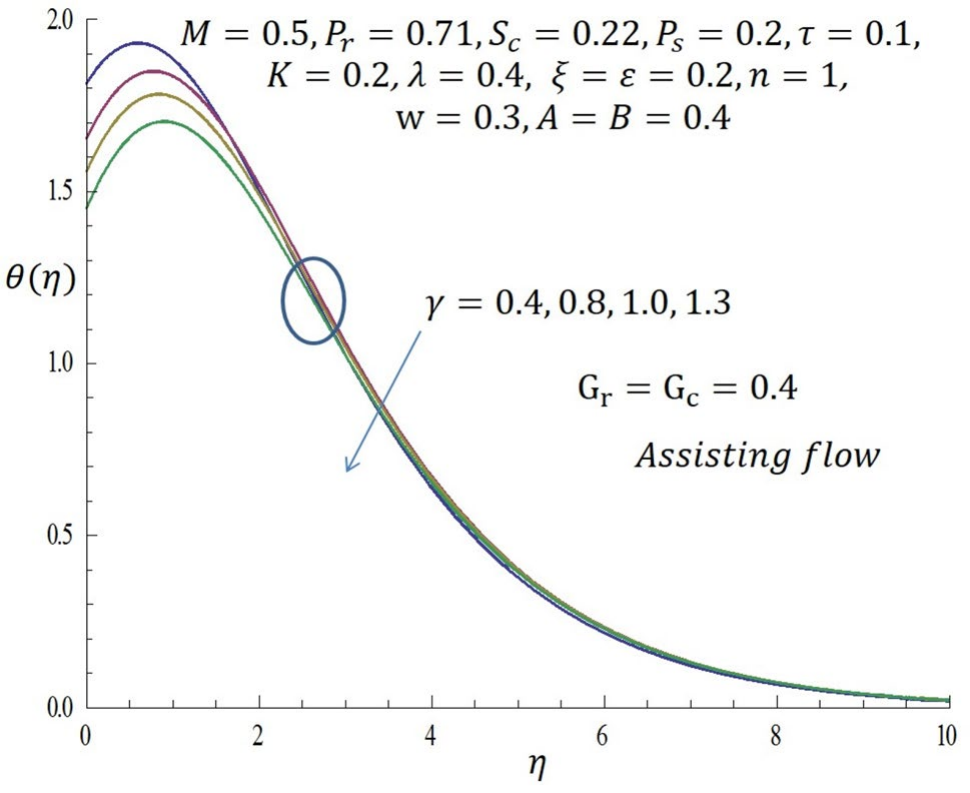
Effect of $\gamma$ on temperature profile when ${G}_{r}={G}_{c}=0.4$.

**Figure 14:**
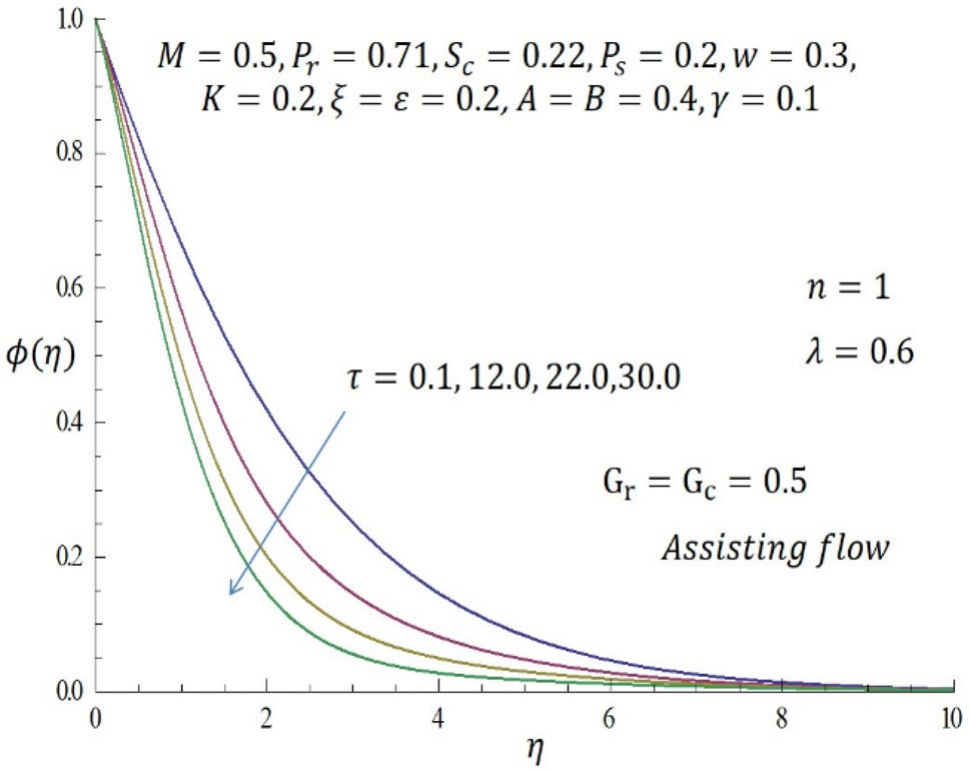
Effect of $\tau$ on concentration profile when $n=1$.

**Figure 15:**
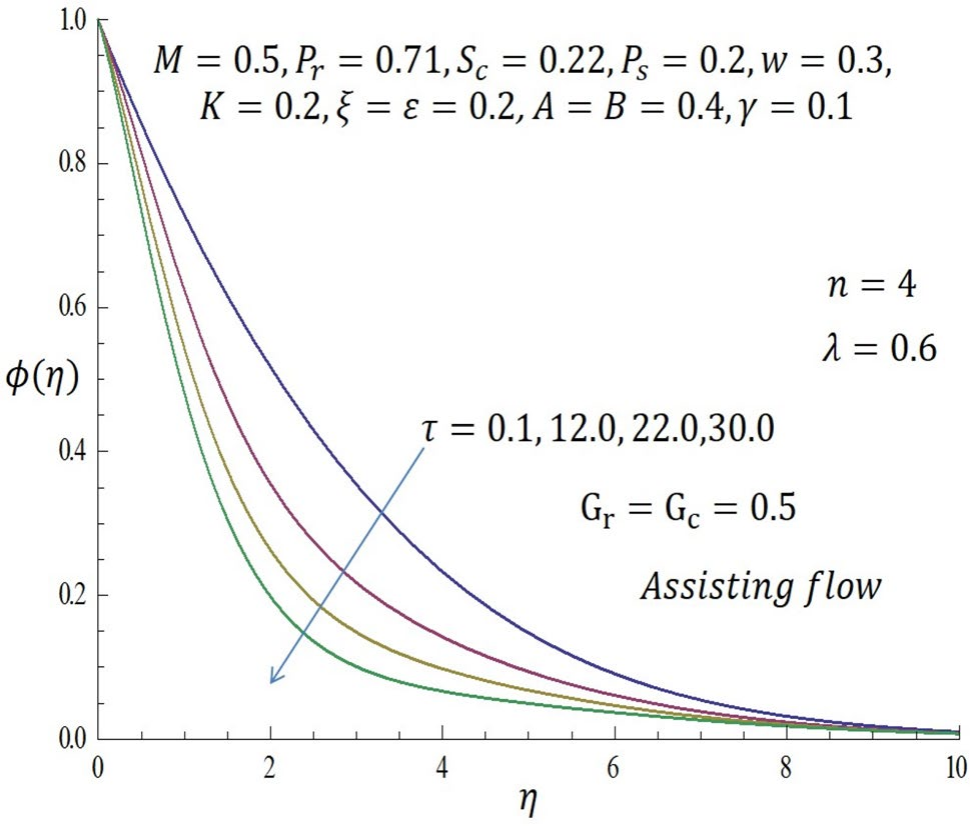
Effect of $\tau$ on concentration profile when $n=4$.

**Figure 16:**
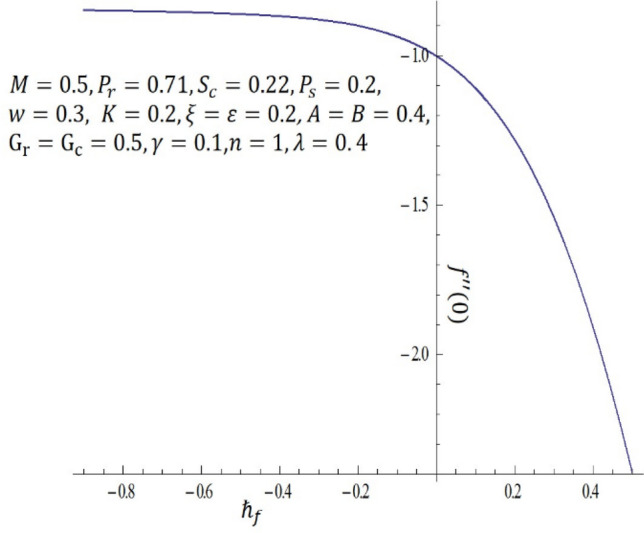
$\hslash$-curve of $f {^{\prime}} {^{\prime}}(0)$ obtained at $10\mathrm{th}$-order of approximation.

**Figure 17:**
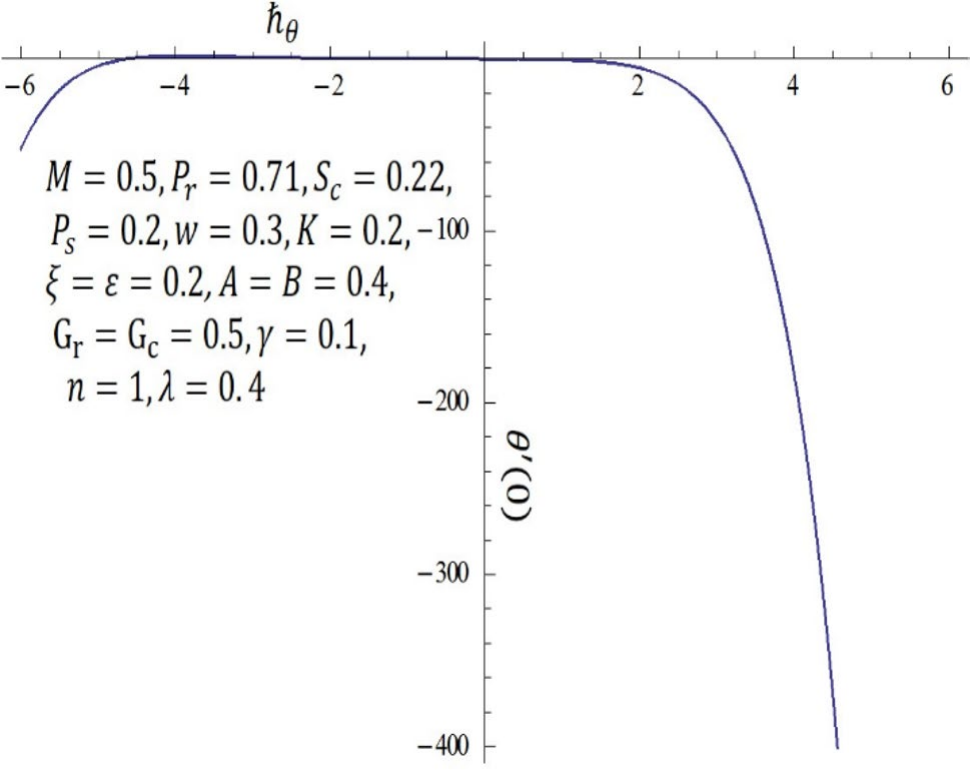
$\hslash$-curve of $\theta {^{\prime}}(0)$ obtained at $10\mathrm{th}$-order of approximation.

**Figure 18:**
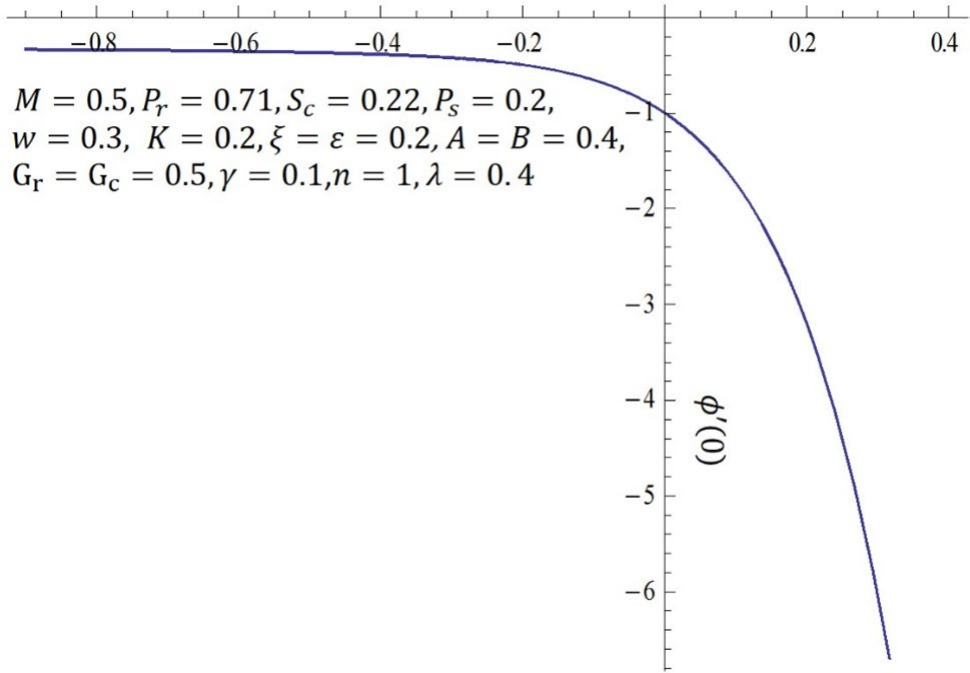
$\hslash$-curve of $\phi {^{\prime}}(0)$ obtained at $10\mathrm{th}$-order of approximation.

Computations were conducted using the approximate analytic method described above for various values of the second grade parameter $K$, temperature dependent viscosity $\xi$ parameter, temperature dependent thermal conductivity $\varepsilon$, concentration dependent diffusivity parameter $\omega$, Biot number $\gamma$, thermophoretic parameter $\tau$, modified buoyancy parameters ${G}_{r },{G}_{c},$ and chemical reaction $\lambda$. Table 1 reveals the numerical values of skin friction coefficients, reduced Nusselt number and reduced Sherwood number of $\xi$ when other parameters are fixed. It is observed that for the first two entries of $\xi$ there is a slight decrease in skin friction coefficients and for the last two entries of $\xi$ there is a significant decrease in the skin friction coefficients while there is increase in reduced Nusselt number for all entries of $\xi$. Also, for the first two and last two entries of $\xi$ there is decrease in the reduced Sherwood number. Table 2 depicts the numerical values of skin friction coefficients, reduced Nusselt number and reduced Sherwood number of $\varepsilon$ when other parameters are fixed. For the first two entries of $\varepsilon$ there is increase in coefficient of skin friction and likewise for the last two entries of $\varepsilon$ there is increase in the skin friction coefficients. Also, there is increase in values of reduced Nusselt number for the first two and last two entries of $\varepsilon .$ Furthermore, it is revealed that there is decrease in reduced Sherwood number for the first two entries of $\varepsilon$ while there is increase in the reduced Sherwood number for the last two entries of $\varepsilon$. Table 3 shows the numerical values of skin friction coefficients, reduced Nusselt number and reduced Sherwood number of $\omega$ when other parameters are fixed. It is observed that the skin friction coefficient is an increasing function of $\omega$, while both the reduced Nusselt number and reduced Sherwood number are decreasing functions of $\omega$. Solutions have been obtained for both assisting flow ${G}_{r}={G}_{c}>0$ and opposing flow ${G}_{r}={G}_{c}<0$. In order to illustrate the results graphically, the numerical values are plotted in Figs. 2, 3, 4, 5, 6, 7, 8, 9, 10, 11, 12, 13, 14, 15, 16, 17 and 18. The effect of the second grade parameter $K$ are presented in Figs. 2, 3, 4 and 5.

**Table 1:** Numerical values of skin friction coefficients for various values of $\xi$.

$\xi$	${P}_{r}$	$K$	$\varepsilon =\omega$	$-{f}^{{^{\prime}}{^{\prime}}}\left(0\right)$	$-\theta {^{\prime}}(0)$	$-\phi {^{\prime}}(0)$
$0.1$	$0.7$	$0.3$	$0.2$	$0.7987$	$0.5701$	$1.0509$
$0.3$	$0.7$	$0.3$	$0.2$	$0.7940$	$0.5728$	$1.0430$
$0.5$	$0.7$	$0.3$	$0.2$	$2.2959$	$0.9100$	$1.5870$
$0.7$	$0.7$	$0.3$	$0.2$	$1.8654$	$2.0338$	$1.0499$

**Table 2:** Numerical values of reduced Nusselt numbers for various values of $\varepsilon$.

$\varepsilon$	${P}_{r}$	$K$	$\xi =\omega$	$-{f}^{{^{\prime}}{^{\prime}}}\left(0\right)$	$-\theta {^{\prime}}(0)$	$-\phi {^{\prime}}(0)$
$0.1$	$1.0$	$0.4$	$0.2$	$3.5899$	$1.3649$	$1.0509$
$0.4$	$1.0$	$0.4$	$0.2$	$3.6302$	$1.4030$	$1.0430$
$0.7$	$1.0$	$0.4$	$0.2$	$1.050$	$0.7630$	$1.5870$
$1.0$	$1.0$	$0.4$	$0.2$	$1.6208$	$1.4064$	$1.1940$

**Table 3:** Numerical values of Sherwood numbers for various values of $\omega$.

$\omega$	${P}_{r}$	$K$	$\xi =\varepsilon$	$-{f}^{{^{\prime}}{^{\prime}}}\left(0\right)$	$-\theta {^{\prime}}(0)$	$-\phi {^{\prime}}(0)$
$0.1$	$1.2$	$0.7$	$0.4$	$0.9910$	$0.8378$	$1.1883$
$0.4$	$1.2$	$0.7$	$0.4$	$1.0433$	$0.8306$	$0.8548$
$0.7$	$1.2$	$0.7$	$0.4$	$1.0718$	$0.8242$	$0.6753$
$1.0$	$1.2$	$0.7$	$0.4$	$1.0902$	$0.8210$	$0.5605$

In Fig. 2, it is observed that as $K$ increases from $0, 0.3$ through $0.7$ to $1.0$ when ${G}_{r}={G}_{c}>0$ (assisting flow), the velocity of the second grade fluid decreases within the domain $0\le \eta \le 3.64$. At the specific region of $\eta =3.68$ all curves merge and decay far away from the heated vertical wall. From Fig. 3, it is observed that incremental values of $K$ correspond to decrease in the temperature of the fluid for the case of assisting flow.

Likewise, from Fig. 4 when ${G}_{r}={G}_{c}<0$ (opposing flow), it is observed that increase in the values of $K$ correspond to decline in the velocity profile near the wall. In Fig. 5, it noticed that the temperature profile decreases as $K$ is increased. The influence of temperature dependent viscous parameter $\xi$ on velocity profile when ${G}_{r}={G}_{c}>0$ is illustrated in Fig. 6. It is clearly observed that the magnitude of the vertical velocity component increases significantly as $\xi$ increases, the velocity boundary layer is developed as the fluid flows over the surface when modified buoyancy parameters ${G}_{r}={G}_{c}>0$. Physically, this observation connotes that in free convection flows bounded by a surface within boundary layer formation on a heated vertical plate, the fluid far from the wall is quite denser than the fluid close to the wall making it possible for the fluid close to the wall which appears lighter to flow at an increased velocity upward towards the quiescent region. Due to this fact, buoyancy forces stimulate a natural convection boundary layer in which the heated fluid rises vertically and it is able to surmount the impact of viscosity. The effect of increasing $\xi$ on wall shear stress is elucidated in Fig. 7. It is seen from the figure that as $\xi$ increases, the wall shear stress also increases. Figure 8 depicts the influence of $\xi$ on velocity profile when the modified buoyancy parameters ${G}_{r}={G}_{c}<0$. The detailed examination shows that as $\xi =b\left({T}_{f}-{T}_{\infty }\right)$ increases from $0, 2.0$ through $4.0$ to $6.0$, when the modified buoyancy parameters take negative value i.e. ${G}_{r},{G}_{c}<0$, the velocity profile increases for larger values of $\xi =b\left({T}_{f}-{T}_{\infty }\right)$. Physically, the observed trend is owing to the fact that there is enough energy necessary to break down the strong intermolecular bonds in the fluid boundary and the viscosity's retarding impact is reduced causing the fluid's velocity to increase.

Figure 9 represents the velocity profile for various temperature dependent thermal conductivity parameter $\varepsilon$ when modified buoyancy term ${G}_{r}={G}_{c}>0$. It is observed that increase in $\varepsilon$ corresponds to increase in the velocity profile. Physically, the heated vertical plate transfers heat energy to the fluid layer very close to the wall thereby causing the fluid velocity to be augmented. Likewise, incremental values of $\varepsilon$ result to enhancement of the temperature of the fluid as revealed in Fig. 10. The effect of variable concentration diffusivity parameter $\omega$ on velocity profile when ${G}_{r}={G}_{c}>0$ is described in Fig. 11. It is observed that as $\omega$ increases, there is a slight increase in the velocity profile. Likewise, from Fig. 12, increase in $\omega$ leads to significant increase in the concentration profile. Figure 13 illustrates the effect of Biot number $\gamma$ on the temperature profile. It noticed that the temperature profile reduces as $\gamma$ increases. The influence of thermophoresis parameter $\tau$ on concentration profile at the first order chemical reaction i.e. $n=1$ when ${G}_{r}={G}_{c}>0$ is illustrated in Fig. 14.

It is observed that the magnitude of the concentration profile decreases with larger values of $\tau$ Physically, thermophoresis is a phenomenon whereby heated particles migrate to a region of diminishing temperature gradient. (i.e. particles move to a region where there is low heat energy).

Likewise, the same effect is observed in Fig. 15 when the 4th order chemical reaction is considered. It is observed that the concentration profile is a decreasing function of $\tau$ even for higher order of chemical reaction.

## Conclusion

The study of the variable thermophysical properties of second-grade fluid flow past a vertical surface with convective boundary condition with special attention to assisting and opposing flows has been studied analytically. The effects of thermophoresis, temperature-dependent viscosity, temperature-dependent thermal conductivity, and variable concentration diffusivity are all taken into account. Using similarity transformation, the governing partial differential equations are converted into nonlinear ordinary differential equations. This investigation yielded the following significant findings:

1. Velocity and temperature profiles are decreasing functions of second grade parameter $K$ for assisting flow and opposing flows.
2. Within the boundary layer, velocity profile is an increasing function of temperature-dependent viscous parameter $\xi$ for both assisting and opposing flows.
3. Both velocity and temperature profiles are increasing functions of temperature dependent thermal conductivity parameter $\varepsilon$ in case of assisting flow.
4. The velocity and concentration profiles increase with increase in concentration dependent diffusivity parameter $\omega$ for assisting flow.
5. For first and higher orders of chemical reaction, the magnitude of the concentration profile decreases with larger values of thermophoretic parameter $\tau$.
